# Comparative genomic analysis of monosporidial and monoteliosporic cultures for unraveling the complexity of molecular pathogenesis of *Tilletia indica* pathogen of wheat

**DOI:** 10.1038/s41598-019-44464-0

**Published:** 2019-06-03

**Authors:** Pallavi Mishra, Ranjeet Maurya, Vijai K. Gupta, Pramod W. Ramteke, Soma S. Marla, Anil Kumar

**Affiliations:** 1 Department of Molecular Biology and Genetic Engineering, College of Basic Sciences and Humanities, G. B. Pant University of Agriculture and Technology, Pantnagar, India; 2 Division of Genomic Resources, ICAR-National Bureau of Plant Genetic Resources, New Delhi, India; 3 Department of Computational Biology and Bioinformatics, Sam Higginbottom University of Agriculture, Technology and Sciences, Allahabad, India; 4 Department of Chemistry and Biotechnology, ERA Chair of Green Chemistry, Tallinn University of Technology, Tallinn, Estonia; 5 Department of Biological Sciences, Sam Higginbottom University of Agriculture, Technology and Sciences, Allahabad, India; 6 Rani Lakshmi Bai Central Agricultural University, Jhansi, India

**Keywords:** Genome assembly algorithms, High-throughput screening

## Abstract

*Tilletia indica* (Ti) - a quarantined fungal pathogen of wheat and its pathogenesis is chiefly governed by pathogen effectors secreted inside the host plant. The *de novo* genome sequencing of several field isolates and stages available could be used for understanding the molecular pathogenesis. The presence of gaps and low coverage of assembled genomes poses a problem in accurate functional annotation of such functions. In the present study attempts were made to improve the Ti draft genome through reconciliation of globally available datasets of three highly virulent monoteliospore cultures of Ti field isolates. It has sequence depth of 107x and N50 scaffold size of 80,772 (more than 26 times as large as achieved in the draft assembly) with highest sequence contiguity, more accurate and nearly complete. Functional annotation revealed that Ti genome contains 9209 genes evolved with many expanded gene families and arranged mostly in a cluster. About 79% of Ti genes were orthologous to other basidiomycetes fungi, Around 7.93% proteins were having secretary signals and 6.66% were identified as highly virulent pathogenicity genes. Using improved Ti genome as a reference, the genomic variation was assessed with respect to repeats, SNPs/InDel, gene families and correct set of virulence associated genes during its life cycle. The comparative intra-species, inter-stage and inter-species genomic variation will have broader implications to understand the gene regulatory networks involved in growth, mating and virulence behaviour of *Tilletia* f. spp. and also for better appreciation of fungal biology and disease management.

## Introduction

Karnal bunt (KB) of wheat crops was first reported in Karnal in India in 1931^1^ and caused by the smut fungus *Tilletia indica* (Ti) basidiomycetes belonging to the subphylum Ustilaginomycotina. Approximately 86 years have been passed since the discovery of the Karnal bunt disease, there is only scanty molecular information is available in relation to the pathogenicity of Ti. It became a major disease which hampers the international wheat trade due to quarantine regulations imposed by several countries^2^. The fungus is heterothallic in nature and undergoes a transition from monokaryotic to dikaryotic developmental stages during its life cycle. Due to heterothallism, there exists large genetic variation amongst pathogen due to the fusion of opposite mating types of sporidia and thus affecting virulence behaviour^3^. During onset of the disease, mating between two compatible allantoids sporidia of Ti and resultant genetic recombination generates significant pathogenic and genetic variability^4^. The primary sporidia give rise to secondary sporidia by budding, which continuously multiply on the host leaf surface^5^ and through monkey jumping it moves from lower to upper leaf surfaces^6^. It infect between the boot leaf and soft dough stage, approximately a fort night, depending on the cultivar and the weather conditions^7^ and later on penetrate the wheat floret through stomata^8^. Growth of the mycelium inside the pericarp eventually raptures the connection between the pericarp and surrounding vascular bundles and as a result, the seed atrophies to varying degree and leading to partial bunt^9^. When the teliospores are liberated at harvest to the soil surface or dispersed on or in grain, the cycle begins again. In the life cycle of Ti, teliospores are the main disease-causing entities, which exist in nature as dikaryons. The teliospores are widely disseminated through the seeds of the host plant inside or outside and soil.

In order to understand the fungal biology of this pathogen, the life cycle has created a rapidly rising demand for development of insights about various components of molecular pathogenesis. Several biochemical and molecular biology approaches have been used for identification and characterization of pathogenic determinants or virulence factors for the elucidation of molecular mechanisms underlying fungal pathogenesis. Availability of complete and accurate genomic information of fungus is required to understand the offence and defence mechanism(s) during wheat – Ti interaction and fungal pathogenesis. To date, attempts have been made by several research groups to sequence and decipher the genome information generated using various next generation sequencing platforms^10–12^. The draft assembly published in 2017 with the total assembly size of 26.7 Mb, was quite fragmented containing over 10,957 contigs whose weighted average (N50) size was 3,009 bp, thus indicating loss of valuable information^10,13^. Redundancy in genetic profiling of the fungus Ti is confusing and necessitates refinement of available genome sequence data generated from assembly of monoteliosporic cultures of Ti. In order to obtain a complete set of coded genes, then it is pre-requisite to acquire the completeness of the whole genome that should be more accurate^14^. The three draft assemblies of the monoteliosporic culture of Ti were used to develop an improved version of genome assembly by using the reconciliation methods. Such strategies have not been utilized in Ti and based on such study; an improved genome sequence of Ti was reported first time^15^ that will help in better understanding of fungal pathogenesis and development^16^.

The improved Ti genome sequence with decreased redundancy and genomes described by different Ti projects was employed to examine inter-stage genomic diversity in teliospores (mycelium), monosporidial lines (+ and − mating types) and dikaryon level during different developmental stages in Ti lifecycle. Such comparative genomic analysis of monosporidial and monoteliosporic cultures will not only help in unravelling the complexity of molecular pathogenesis of Ti pathogen of wheat but also understanding the mechanisms associated with intra-species and inter-species genetic variability at molecular level. Further, the direct comparison between these genome assemblies of Ti was made by the detailed analysis of the whole genomes with monosporidial and dikaryon level of developmental stages, core protein coding genes including secretary proteins analysis and comparison of orthologous gene families led to the identification of pathogenesis-related genes. The diversity of Ti isolates were analyzed using genome based variation analysis of different Ti isolates. Identified SSR, SNP, Indel and repeat elements among different field isolates analyzed apart from establishing accurate phylogenic relationships that may help in better understanding the plasticity and dynamic behaviour of Ti genomes to overcome the host plant immune systems.

## Results

### Improvement of the draft genome through reconciliation algorithms for intra-species monoteliosporic assemblies of different isolates

#### Refinement of the fragmented draft genome of TiK isolate through Hybrid *de novo* reassembly approach

Genome assemblies using hybridSPAdes performed with multiple k-mer combination ranging from 21 to 101 suggested that the best assemblies was built with k-mers 63, 65, 67 and 69. It showed the least fragmented sequences, least number contigs with high N50, mean and median scaffold length. The hybrid assembly for TiK from both illumina and Pacbio reads resulted in 3,727 scaffolds with the N50 length of 32,961 bp.

#### Draft genome merging of three different monoteliosporic isolates

Metassembler reconciliation algorithm includes genome reconstruction process. To circumvent this, we simulated three genomes merges and optimize all three monoteliosporic genome assemblies and gave the best assembly after systematically evaluating the number of permutations of merging using the compression-expansion (CE) statistics. The number of internal gaps (consisting of Ns) inside the scaffolds was filled using mate pair information. The remaining gaps were filled by searching unique contig end sequences against unincorporated reads. We observed that filtering of repeat elements led to significant reduction of number of gaps and prediction of increased number of pathogenicity related genes. The new merged assembly had 787 lowest number of scaffolds with high contiguity (Table 1) and coverage depth of 107x.

**Table 1:** Whole genome assembly features of different monoteliosporic field isolates of *T*. *indica*.

Features	TiK_1	TiK	RAKB_UP_1	DAOM 236416
Number of scaffolds	787	3727	1736	1666
Minimum scaffolds length	509	500	500	969
Maximum scaffolds length	4,84,720	1,23,819	4,62,469	3,02,251
N50 (bp)	80,772	25,879	58,667	82,468
File size (Mb)	31	31.55	33.77	30.16

#### Improvement and estimation of quality of assembly for its completeness

The improved sequence has achieved the maximum scaffolds length and lowest the number of scaffolds as compared to other monoteliosporic sequences (Table 1). The BUSCO evaluation of completeness of the conserved proteins in the assembly of the *T*. *indica* genome sequence predicted that it was 97.2% complete. A total 1,438 BUSCO groups were searched, the genome assembly found to contain 1,397 complete single-copy BUSCOs, 18 complete duplicated BUSCOs, 9 fragmented BUSCOs, and 14 missing BUSCOs. Genome assembly graph complexity was reduced as sequence length increases. De Bruijn graphs for *T*. *indica* TiK isolate resulted non-branching paths have been collapsed with increasing the k-mer size and significantly simplified the graph. Assessment of genome assembly with the FRC method tends to the result that our genome assembly gave better FRCurve than the other datasets assemblies, which suggests that the continuity of our assembly is acceptable. All of these assessment statistics revealed that our improved genome sequence has high contiguity, accuracy, and more importantly a high degree of gene space completeness for effective gene prediction.

#### Gene pool analysis of three monoteliosporic assemblies with respect to improved Ti K_1

**Figure 1:**
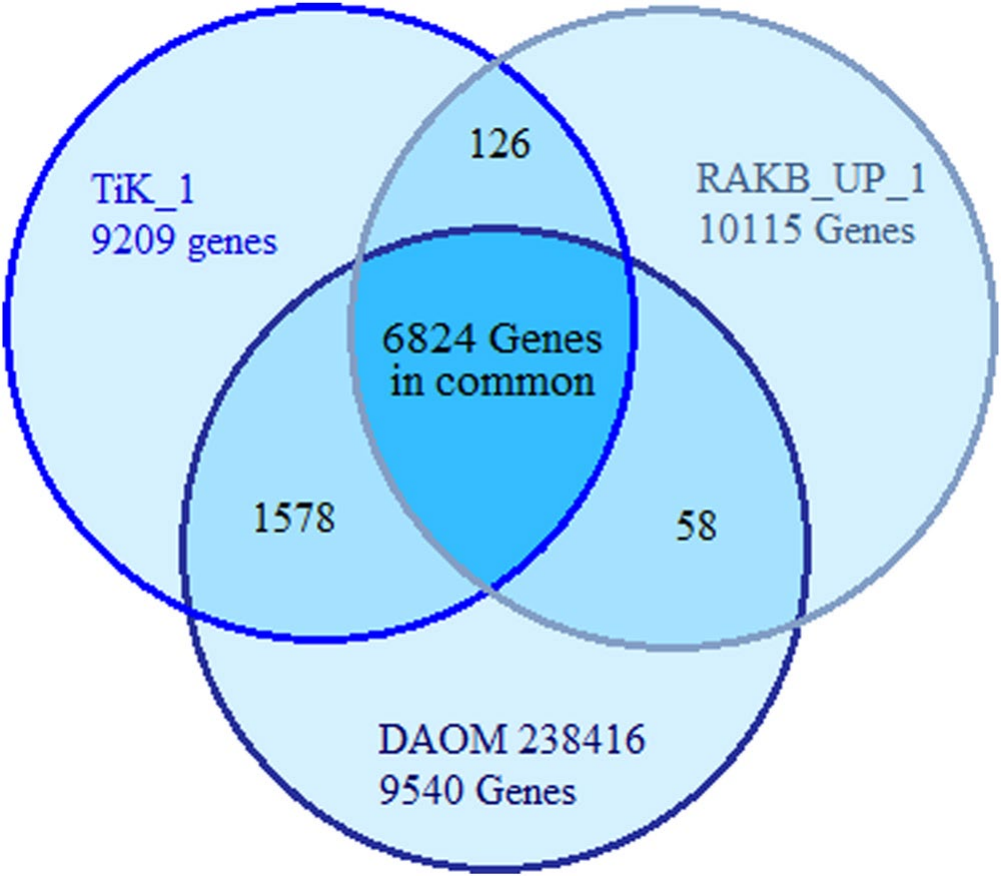
Overlap among the gene sets of *T*. *indica* monoteliosporic isolates indicating the presence of core genes.

Comparative gene prediction of all monoteliosporic assemblies included in this study revealed different numbers of gene count. Large numbers of gene count were found with highly fragmented assemblies. Improved dataset goes down in total gene count shows decrease in redundancy in genome sequence compared to the another dataset. The comparative gene coverage statistics of four genome assemblies were 9209 for TiK_1, 11535 for TiK isolate, 10115 for RAKB_UP_1 and 9540 for DAOM 236416 https://www.uniprot.org/proteomes/UP000077521/. Total of 6824 core genes was present among all the three isolates included in this analysis as suggested in the venn diagram in Fig. 1.

#### Genome annotation statistics of the improved draft genome of TiK_1 isolate

Gene identification from the TiK_1 dataset against the *Ustilago maydis* as a model suggested 9209 total counts of genes in an improved version, which showed a low number of finding comparatively with another dataset which was included in merging and were used to get improved one.

#### Identification of repetitive sequences and transposable elements in the improved draft genome of TiK_1 isolate

A total number of 548 elements were found as simple repeats of length 65,869 bp and 23 of low complexity repeats of 3,869 bp found to improved sequence. Identified transposable elements were 1,877 in number out of which 573 were found as gypsy with the highest count, followed by 309 times the cacta.

### Intra-species, inter-stage and inter-species genome variations amongst different isolates, development stages and species of *Tilletia*

#### Gene orthologies based variation

**Figure 2:**
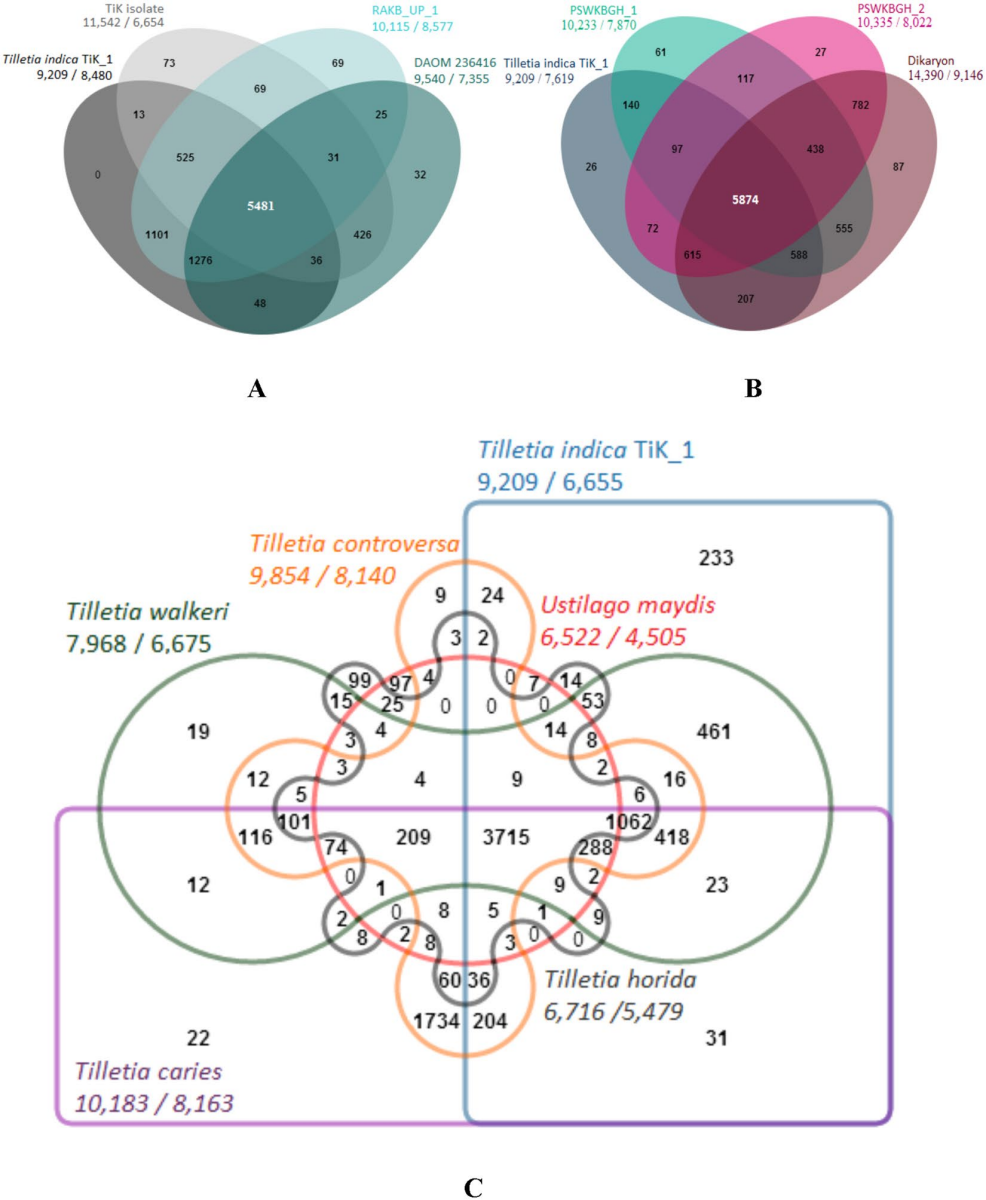
Venn diagram showing the overlap of orthologous genes found within the genus *Tilletia*. (**A**) Intra-species proteome variation amongst different monoteliosporic isolates describing a number of common and unique protein clusters. (**B**) Inter-stage proteomic variations during fungus development showing comparative protein clustering within three developmental stages of *T*. *indica*. (**C**) Six-way venn diagram is showing the inter-species distribution of shared gene families (sequence clusters) among closest basidiomycetes phytopathogenic fungal genomes (number of clusters are shown under each species name as the Total number of genes/number of clusters).

Phylogenies are important for addressing various biological questions such as relationships among species as well as genes, the origin and spread of pathogen and demographic changes amongst different *T*. *indica* isolates and genus *Tilletia* f. spp. However, the formation of orthologs are the key steps in finding gene evolution, we identified unique and shared gene families and proteomes among intra-species monoteliosporic isolates, inter-stage and among the different species of *Tilletia*. When sequencing efforts include more than one genotype, an unsuspected level of structural variation is may found. Intra-species variations found to be about 77% of shared gene orthology among monoteliospore genomes (Fig. 2A). From monoteliosporic to monosporidial stage of development 79% genes were shared, from monosporidial to dikaryon shared genes were 72% and from dikaryon to monoteliosporic stage 71% genes were shared (Fig. 2B). Further, comparison of a contiguous region of the species of *Tilletia* revealed that 21% of the sequences were not shared. Comparison of *Tilletia indica* https://www.ncbi.nlm.nih.gov/assembly/GCA_002997305.1/, *Tilletia caries* https://www.uniprot.org/proteomes/UP000077671/, *Tilletia horrida* https://www.ncbi.nlm.nih.gov/assembly/GCA_001006505.1/, *Tilletia walkeri* https://www.uniprot.org/proteomes/UP000078113/, *Tilletia controversa* https://www.uniprot.org/proteomes/UP000077684/ and *Ustilago maydis* https://www.uniprot.org/proteomes/UP000000561/ proteomes revealed 3,715 genes clusters in common to all six *Tilletia* f. spp. (Fig. 2C) and thus it may be representing ancestral gene families. In brief, the *Tilletia* f. spp. form 9,314 clusters, out of which 8,835 orthologous clusters (at least contains two species) and 3,307 single-copy gene clusters. A total of 6,655 gene families were identified in the TiK_1 genome, among these 233 were unique gene families. Comparisons of orthologous genomic sequences from multiple *Tilletia* f. spp. can reveal numerous genic rearrangements with respect to gene insertions, deletions, duplications or translocations.

#### Phylogenetic variation

**Figure 3:**
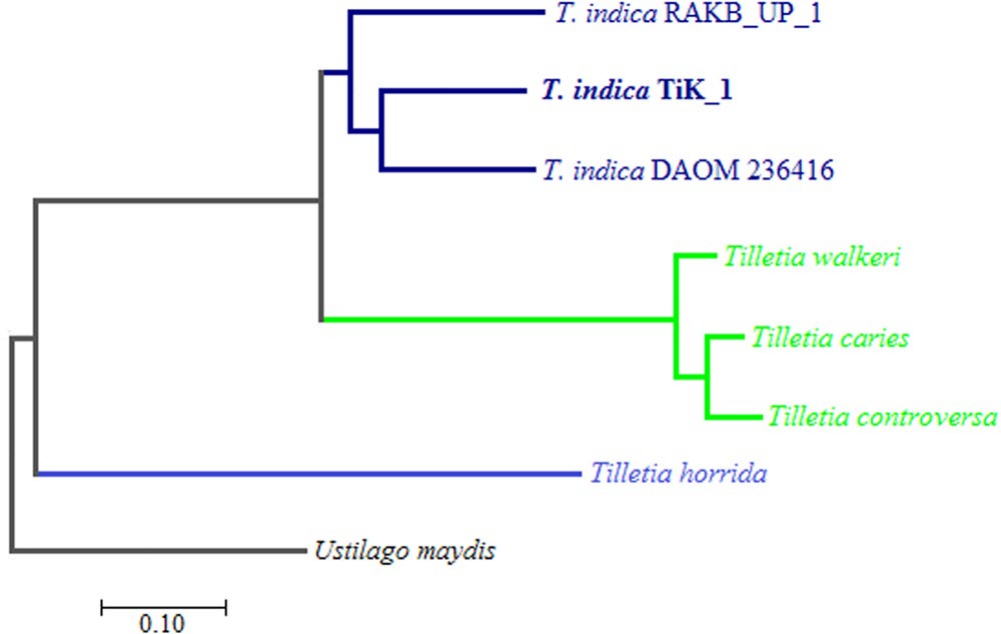
Phylogram is describing phylogenetic profiling of intra-species and inter-species phytopathogenic *Tilletia* spp. along with basidiomycetes fungi.

Phylogenetic profiling of *T*. *indica* with other phytopathogenic basidiomycetes fungi involves the comparison of phylogenetic data across gene families. Comparison constructed the patterns genetic relationships among *Tilletia* spp. and among another basidiomycetes fungus. Phylogenetic relationship of this sp. was inferred from proteome level profiling. Phylogenetic tree grouped into two clades; one is grouped to smut fungi (*U*. *maydis*) separately as an out group and second is grouped to bunt fungi (all *Tilletia* sp.). Gene families of *Tilletia indica* TiK_1 was found nearly correlated to *Tilletia indica* DAOM 236416 isolate followed by the *Tilletia indica* RAKB_UP_1 (Fig. 3). However, the phylogenetic variation among *Tilletia* f. spp. in second sub-clade proofs coupled evolution and the functional relation among them except *T*. *horrida* which placed in second sub-clade.

#### Repetitive sequences and transposable elements based variation

Repeats elements may be the source of genome variation and rapid adaptation to different hosts and environmental condition. However, repeats are the most frequently occurring region in case of the eukaryotic genome but in case of fungal genomes very less amount of repetitive sequence occurs as compared to other eukaryotes which are very rarely exceeds 5% of the genome. Evidence for this we get when, identification of repeats using Repeat-masker in *Tilletia* genomes account for only about ~0.20% of the total genome. The percentage observed in this study is quite a consistent number of repeat sequences along with other *T*. *indica* isolates. However, higher variations were observed in *Tilletia* f. spp. indicating the role of such sequences and elements in the speciation process.

#### Microsatellites based variation

Microsatellites or SSRs markers are extremely helpful for molecular recognition, genetic differentiation among individuals and populations in fungi^17^. The whole genome wide identification for SSRs in *T*. *indica* genomes included in this study was done in order to collect genomic resources for population characterization. Scanning of 32.78 Mb *T*. *indica* genome sequences revealed the presence of total 5,734 SSRs. Of which, 5536 were simple and remaining 198 were complex types. Trinucleotides repeats were the most abundant occupying 42.71% of total SSRs, followed by 28.13% dinucleotides (1613), 22.82% mononucleotide (1309), and 2.87% tetranucleotides (165) repeats. The remaining SSRs were a complex type, with 0.97% of penta and 2.47% of hexa nucleotides.

**Figure 4:**
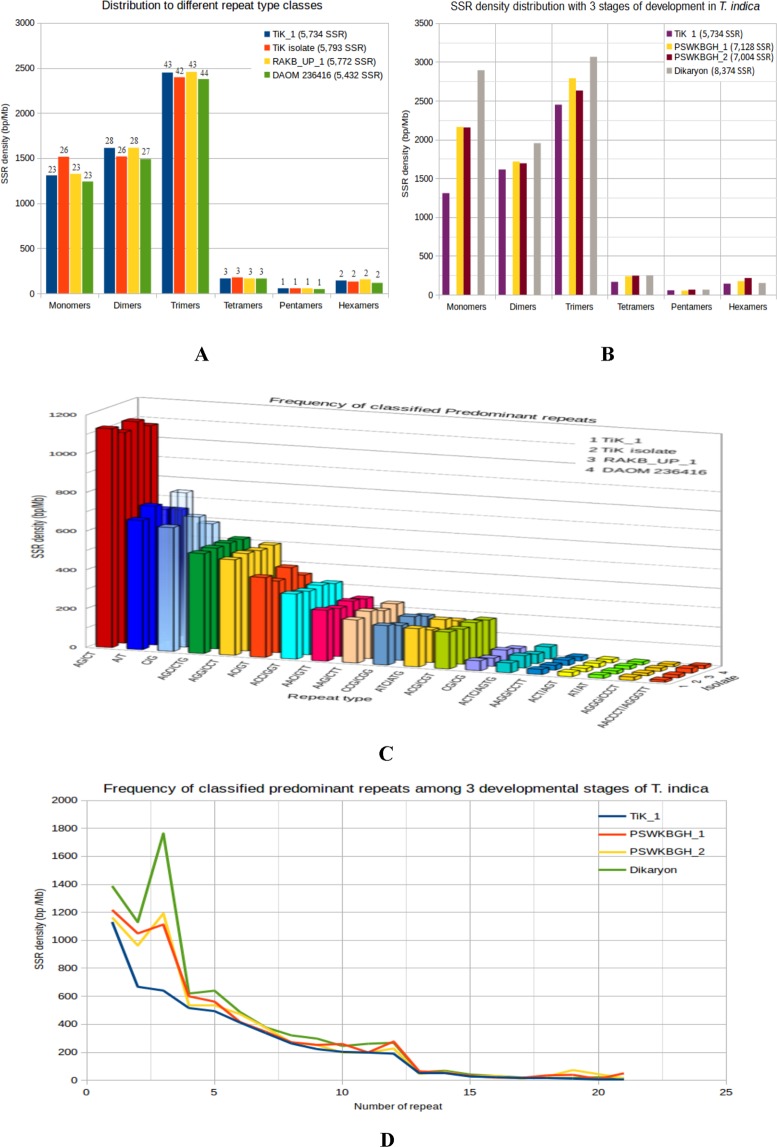
SSR density in different *T*. *indica* isolate genome. (**A**) Explaining the distribribution of monomers, dimmers, trimers, tetramers, pentamers and hexamers among four *T*. *indica* isolates. (**B**) The comparison of mono-, di-, tri-, tetra-, penta- and hexamers among three developmental stages of *T*. *indica*. (**C**,**D**) The frequency of the most common motif appears predominatly is displayed for each developmental case (Additional File 1).

Among 1309 mononucleotide repeats, the mononucleotide motifs exhibited a strong bias towards 51.03% of A/T repeats compared with 48.96% of C/G repeats type. Among 1613 dinucleotides microsatellites, AG/CT type (70.11%) of microsatellites were most common type in the genome followed by AC/GT type (25.54%), and CG/CG type (3.22%). The AT/AT type dinucleotides microsatellites were present at a very low proportion (1.11%). In trinucleotide SSRs repeats (2449), around 13.76%, 10.73%, 9.10%, 8.28% of SSRs were of ACC/GGT, AAC/GTT, AAG/CTT and CCG/CGG types, were most abundant respectively. Among the other types of repeats, the AAT/ATT type was lowest (0.12%) in the genome of *T*. *indica*. The poor distribution (2.87%) of tetranucleotides microsatellites was present in the genome of *T*. *indica*. Maximum number of predominant SSRs repeats were of AG/CT type followed by A/T, C/G and AGC/CTG among three developmental stages. The overall analysis showed that the relative abundance of tetra, penta and hexa SSRs types were low as compared to mono, di and tri SSR types in *T*. *indica* TiK_1 genome sequences. The similar way of observation was performed in other *T*. *indica* isolates and we find SSR length variation between different isolates, were represented in Fig. 4A,B. However, the overall density of SSR was found to be high as trimers. Such repeats found in this study will have immense importance in genomic organization and function of *T*. *indica* and it may be associated with disease conditions, their systematic analysis has not been reported. In future, the detailed analysis may provide a new mechanism for genotypic variation between strains by which species primers must be synthesized complementary to such flanking regions, followed by amplification and polymorphism testing for genotyping.

The pathogen may increase the repetitive elements and have an important role in duplications of the genome (expansion of gene families, especially for virulence or increased pathogenicity), which helps to produce those enzymes, proteins which increases their survival and pathogenicity^18^. The analysis of variation in repeats reflects the number of predominant repeats was increasing during the development stage of fungal lifecycle which proceeds from haploid to diploid stages. Such repeats are predominantly increased after fusion of opposite mating types i.e from the haploid monosporidial (+)ve and monosporidial (−)ve to the fusion of mating types to form dikaryon (Fig. 4D**)**. The enhancement of such repeats during disease cycle of KB pathogen provides an advantage to the pathogen as it may help to produce those proteins during infection with in host and thus increase their survival and pathogenicity. The increase in repeats during disease cycle alters the molecular chemistries involved in host modifications thus better growth and development of fungal pathogen. We observed that filtering of repeat elements led to significant reduction of number of gaps and prediction of increased number of pathogenicity related gene. There might be competitive exclusion among *T*. *indica* sps. due to evolutionary change or behavioural shift of traits according to geographic distribution.

#### Open reading frame based variation

Each node or contig is showing a number of total ORFs, just by adding all ORFs of different nodes we get the total number of ORFs for the genome. We found total count of 4,75794 ORFs in TiK_1, 5,72,341 and 5,67,880 in two monosporidial lines PSWKBGH_1, PSWKBGH_2 respectively, followed by 6,61937 for PSWKBGD_1_3 dikaryon sequence. By analysing the ORFs we can predict the possible correct amino acids that are producing during the translation process. It identifies the all open reading frames or the possible protein coding region in sequence. So, here the prediction of the correct ORF from newly assembled improved genome sequence is important for validating the correct length of a gene found and for a further finding of promoter regions. This is an important step required for our wet lab experiment based validation like PCR sequencing and primer design etc.

#### Frequency distributions of variants

We examined genome-wide variations in TiK genome sequence and as a result, 37,906 SNPs in TiK_1 genome sequence, with 26.8% in coding regions, and 47,329 SNPs in TiK isolate, with 26.3% in coding regions, were identified. TiK_1 and TiK isolates had a similar SNP pattern of distribution regions. There were 2,571 InDels identified in TiK_1 and 2,683 identified in TiK isolate. The significantly fewer InDels may be a result of lower sequencing depth. Interestingly, the distribution pattern of InDels was different from the SNP pattern. Although SNPs and InDels in coding regions can have effects on associated proteins and sometimes alter the phenotype, the chance and level of the influence from SNPs or InDels are hard to predict. Nearly linked SNPs to coding regions may have some impact on future marker development to develop resistant variety of wheat. Thus, it is suggestive that SNPs containing regions were most polymorphic, which causing large modification in protein sequences and thus altering gene function, which can give the evidence of pathogenesis in the future examination. The KB pathogen undergoes the rapid genetic recombination during development that leads to several mutations. However, many of the mutations that are not found in coding regions or within actual genes, which makes it difficult to understand their role in alteration of function but certainly involved in huge genetic variation. Using the SNP data from our study associated with genes, we will be able to find the altered behaviour of fungal disease causing ability more quickly. This will help in understanding the biological questions that why some particular genes induced virulence as KB.

### Genome annotation for elucidating the functions

#### Functional characterization of the improved draft genome sequence of monoteliosporic TiK_1 isolate

In predicted genes, the total hits were 5692 out of which considerable proteins with high similarity were 3793, which is functionally annotated and 353 were having no hit in the non-redundant database against 6788 proteins of *Ustilago maydis*. 1260 proteins were annotated as hypothetical or putative proteins. The other feature based functional annotation hits with the other available database is represented in Table 2.

**Table 2:** Statistics for functional annotation showing gene content of *T*. *indica* TiK_1.

Feautures	Numbers	Percent (%)
Swissprot	4,890	53.10
Uniprot-isoform	3,185	34.58
Genes with signal peptides	1,209	13.12
Transmembrane proteins	436	4.73
Unannotated	4,642	50.40
Annotated	3,793	41.18
KEGG pathway	2,519	27.35
Panther class protein	2,052	22.28

#### Protein family classification

**Figure 5:**
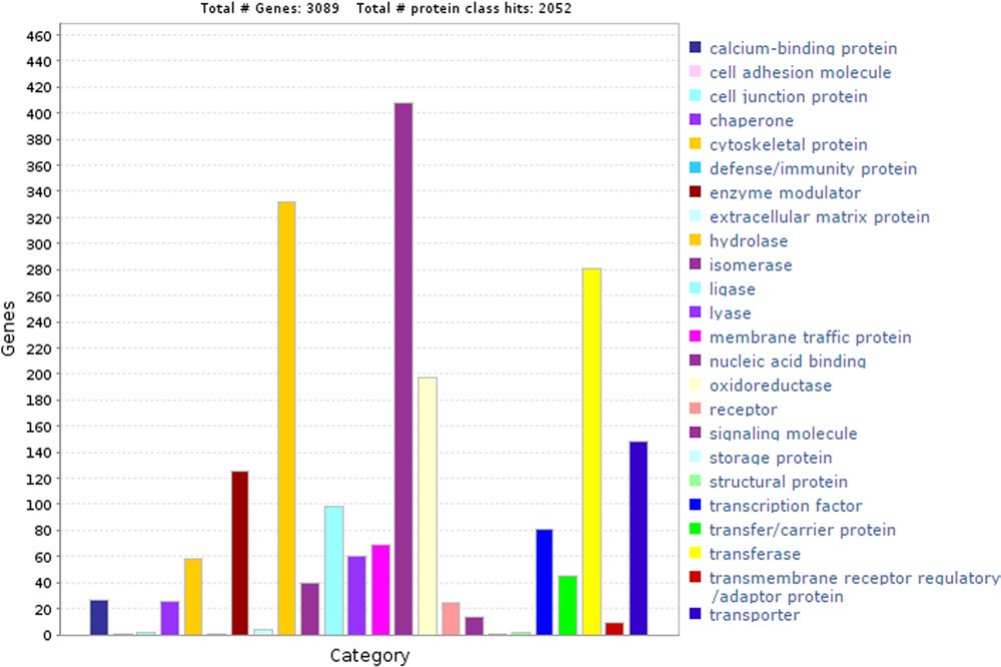
Protein family annotation shows the distribution of most abundant protein class assigned to *T*. *indica* genes.

We classified the proteome of *T*. *indica* into different class of protein families based on sequence similarity with the 6,377 annotated protein class for 6,788 gene list of *U*. *maydis*. In rapidly evolving *T*. *indica* genomes, we found that a significant number of identified genes belonging to functions vital for pathogen survival and successful infection. Among the 9,209 genes with at least mapped to one protein class, only 2,052 were annotated and categorized into 24 protein classes (Additional File 2), majority of this belonging to hydrolases (glucoside hydrolases) and transferaces (glycosyl transferases) as shown in Fig. 5, and these having main role in pathogenesis.

#### Identification of pathogenicity genes implicated in virulence

In order to find the plant pathogenic fungi two computational pipelines were linked together to filter out the final set of pathogenicity genes. Genes involved in pathogenicity mainly depends on or having direct connections with the secreted proteins. In context with finding secreted proteins, secretome analysis revealed a total number of secretory proteins in the *T*. *indica* genome. Total of 731 (7.93%) SignalP proteins were assigned as secretory proteins and 1204 (13.07%) target proteins were involved in the secretory pathway and these protein sequences contained signal peptides. We proceed through the proteins of combined Signalp and TargetP predicted proteins, having secretory signals (Additional File 3). The duplicates were removed from predicted secretory proteins. Further, these secretory proteins having the presence of total 456 trans-membrane domain, suggested that secretory proteins were zero transmembrane domain (TmHmm 0), 185 proteins had one transmembrane domain (TmHmm 1) including 23 highly probable (GPI) anchor containing sequences. The growth efficiency and aggressiveness of fungal pathogens are often linked with their carbohydrate active enzymes and these enzymes required for degrading plant cell walls is a crucial factor for pathogen invasion. The secretory proteins having secreted carbohydrates enzymes were found abundant with Glycosyl Hydrolases (GH) families and Glycosyl transferases (GT) families followed by carbohydrate esterases (CE) families. These found glycosyl hydrolases and glycosyl transferases having direct role in degradation of plant cell wall for pathogen growth. Genes in other isolates included in this study were analyzed using the same methods for comparative analyses.

Proteins of the secretory pathway carry a targeting sequence in their precursor protein sequences and are transported co-translationally across the Endoplasmic Reticulum (ER) membrane. Proteins in the ER are further transported into the Golgi apparatus, plasma membrane, lysosome, vacuole or the extra cellular space. These signal proteins were confirmed with N-terminal targeting sequences that they are involved in secretory pathways. Integration of these proteins with the pathogen-host interaction proteins discriminated prediction of the final set of pathogenic genes.

**Figure 6:**
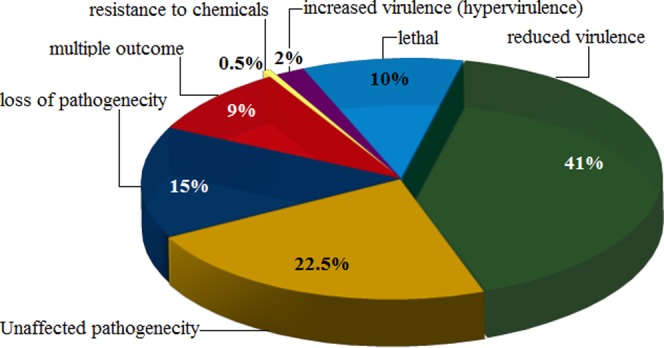
Virulence associated genes (VAGs) belonging to *T*. *indica* genome sequence producing high-scoring segments pairs found with 80% of sequence similarity, 40% of sequence identity with e-value of 1e-6.

To find potential virulence associated genes, a whole genome BLAST analysis conducted against the pathogen-host interaction (PHI) gene database revealed a collection of genes as pathogenicity proteins. After removing the genes that were not related to pathogenicity, we identified 614 putative virulence associated genes (VAGs) in *T*. *indica*. Out of which, total of 82 genes were related to loss of pathogenicity, 10 genes to increased virulence, 49 genes to lethal and 2 genes resistance to chemicals. With high-scoring sequence similarity, we found only 220 genes as VAGs (Fig. 6) (Additional File 4). Further, these pathogenicity-related genes were analyzed for functional characterization. Comparative genomics of *T*. *indica* isolates of different agro-climatic zones reveals the expansion of different outcomes of pathogenic genes as virulence associated genes (VAGs) in Karnal bunt disease (unpublished data). The most plausible explanation for this is due to the differential pathogenic response to different host species of wheat.

#### Functional classification of virulence associated genes (VAGs)

**Figure 7:**
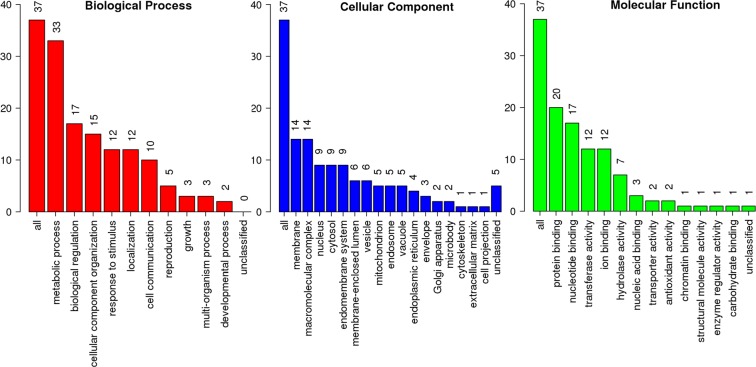
Functional annotation of PHI accession assigned to VAGs based on gene ontology (GO) categorization. Some VAGs may be matched to multiple GO terms.

The PHI-base accessions assigned to *T*. *indica* genes revealed, the majority of fungal pathogenesis genes hits were assigned to *Fusarium graminearum* followed by *Magnaporthe oryzae* and *Ustilago maydis* (Additional File 5). Many of these accessions are species-specific proteins. Further, 220 *T*. *indica* genes classified as virulence were GO classified and gene ontology terms were assigned to 51% of PHI-base accessions as biological process. In the biological process category, the metabolic processes (29.5%) were most highly represented (Fig. 7). For a total of 49% (108 proteins), no GO annotation could be made. Most of the hits with *U*. *maydis* were associated with loss of pathogenicity followed by reduced virulence. Genes assigned as miRNA target were 48, 54 genes as transcription factor target, 7 genes in signal transduction, 5 as mitogen activated proteins (Hog1, Glo1, Cln1, Rho1 and Gpa1 as MAPK). Further, found *T*. *indica* species species proteins could be model for the identification of the fungal pathogenic determinants which can serve as new molecular targets for fungicide development, novel biomarkers for the development of diagnostic tools.

#### Identification of candidate VAGs involved in molecular networks and pathway of pathogenic response

**Figure 8:**
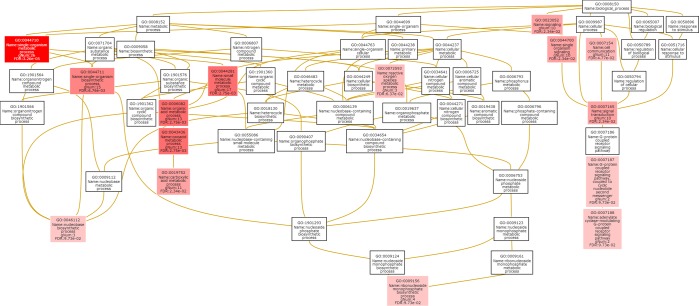
Enrichment network graph for VAGs based on gene-ontology domains (biological process, cellular component and molecular function) in terms of neighbouring of GO terms or GO term overlap.

The overlap between the biological pathways of VAGs in pathogenic response was analysed using all VAGs against KEGG database. We identified a total of 84 genes involved in biological pathways with low false discovery rate (FDR). Gene set or functional enrichment analysis (GSEA) helps to identify classes of genes or proteins that are over-represented in a large set of genes or proteins and it may have an association with disease phenotypes. Gene ontology enrichment analysis or Mapping of genes to KEGG pathway on the basis of GO terms revealed various genes being in the pathway, which are associated with each other were grouped together in relevant pathways that are over-represented in the dataset. This approach typically examines whether a group of related proteins in the same sub-cellular compartment at the same time, indicating there should be such interactions could happen. Results suggest set of grouped genes of *T*. *indica* VAGs (Fig. 8**)** are useful for identifying regulatory events that influence multiple biological processes and pathways.

## Discussion

Pathogen interactions are dynamic and involve high rates of crossing resulting in increased pathogenicity and enhanced capability to resist the host plant immune system. In this study, meta-assembly and other next generation sequencing techniques^19^ are employed to obtain improved *de novo* hybrid assembly of *T*. *indica* field isolate (TiK) using two publicly available isolates of monoteliosporic *T*. *indica* genome assemblies. We developed first time the improved version of *T*. *indica* genome and reduced the redundancy in reported draft genomes of *T*. *indica*. Genome variation estimated at 3 stages of development of teliospores revealed significant genomic diversity amongst monoteliosporic, monosporidial and dikaryon especially in terms of repeats and gene complexity. Comparison of genomic data obtained from *T*. *indica* isolates, reveals conserved and varied molecular machinery underlying the infection capabilities of the fungus. Predicted isolate-specific genes, SNPs, InDels and SSRs and repeat regions among different species in the present study of genus *Tilletia* fungi may help wheat breeders in genome-wide variation analysis. Identified core genes from proteome analysis of different isolates may aid in field selection. Results from comparative gene orthology and phylogenetic profiling among the analyzed *Tilletia* isolates and identified core and orthologous gene families of *T*. *indica* during different developmental stages further enriched our current understanding about gene pool and its diversity. The reported improved sequence of *T*. *indica* would be a rich platform for additional plant-pathogen studies. Even though this is the fourth genome sequence, we observed presence of wide diversity among analyzed field isolates of *T*. *indica* genome. In particular, the repertoire of pathogenic genes having many variations among these isolates may help to explain some of the observed pathogenicity-related phenotypic variations, thus opening the way to design of new control methods for control KB disease in field conditions.

Availability of finished, improved and more accurate genome sequence, reported variance and comparative genomic studies existing among closely related *Tilletia* spp. will provide a rich resource to fungal biological studies aid the research community of fungal biology and Karnal bunt in particular. Near-complete and non-redundant genome sequence available from this study will be used for devising effective crop protection and successful breeding or genetic engineering strategies as part of development of resistant wheat cultivars showing immunity against KB fungus^20^.

## Methods

### Retrieval of *T*. *indica* fungal datasets

Recent whole genome sequencing projects have facilitated the different *T*. *indica* genome assemblies. The complete set of standard whole genome sequences for the Karnal bunt fungus *T*. *indica* with respect to intra-species monoteliosporic isolates, inter-stage monosporidial and dikaryon lines were obtained from the National Center for Biotechnology Information (NCBI) database (https://www.ncbi.nlm.nih.gov) to the local storage, having assembly accession numbers GCA_001645015.1, GCA_002220835.1, GCA_001689995.1, GCA_001689945.1 and GCA_001689965.1 for the isolates DAOM 236416^11^, RAKB_UP_1^12^, PSWKBGH_1, PSWKBGH_2^21^ and PSWKBGD_1_3 respectively. A wide range of basic statistics was considered for each assembly and given below in Table 3.

**Table 3:** Statistical description of *Tilletia indica* genomes sequencing projects.

Isolates	Datasets	Genome length (bp)	N_50_	No. of scaffolds	Coverage
monoteliosporic	TiK isolate	26,707,738	3,009	10,9 57	162X
	DAOM 236416	30,384,772	82,468	1,666	176X
	RAKB_UP_1	33,771,691	58,667	1,736	99.91X
monosporidial	PSWKBGH_1	37,460,344	2,00,513	366	388X
	PSWKBGH_2	37,216,861	1,32,740	470	400X
Dikaryon	PSWKBGD_1_3	4,37,36,635	12,288	8,805	139X

### Preprocessing of raw sequence reads

The Illumina and PacBio sequence reads of TiK (*Tilletia indica* Karnal) isolate^10^ were quality checked using FASTQC v0.11.5 (http://www.bioinformatics.babraham.ac.uk/projects/fastqc) and fastQValidator v0.1.1 (https://github.com/statgen/fastQValidator). Removal of contaminated reads was performed to get the error corrected reads. The Lower quality bases with Phred quality score of less than Q30 (base calling accuracy with less than 99.99%) and the adapter sequence contamination in raw reads were removed using PRINSEQ v0.20.4 (https://sourceforge.net/projects/prinseq) and repaired the reads using BBmap v37.66 (https://sourceforge.net/projects/bbmap).

### Improvement and assembly reconciliation

**Figure 9:**
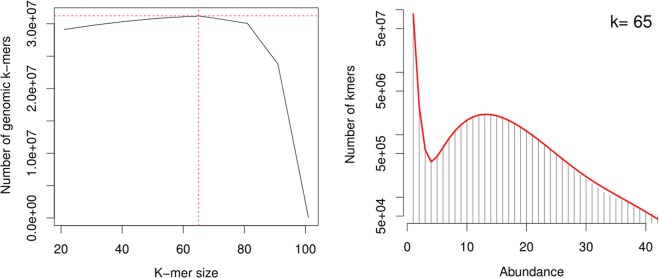
The histogram model to estimate best K-mers with given frequencies suggested for the TiK dataset.

Contigs were *de novo* assembled with the high-quality error corrected Illumina and PacBio reads using hybridSPAdes v3.11.0^22^ and data is provided in Table 4, which is based on the de-Bruijn graph approach, with a highest accuracy. A seed value of 13 was used (t parameter) and a minimum of 10 pairs were required to join contigs (n parameter). HybridSPAdes collects the information generated from fix-length words of kmers shared by overlapping reads. So, initially multiple genome assemblies were generated with multiple k-mer combinations in the range between 21 to 101 and assessed assembly quality statistics such as N50, maximum contig length, number of contigs in the assembly and the total amount of bases in the assembly^23^. Kmergenie^24^ sums the predicted number of genomic kmers over all abundances under the histogram curve. A kmer length of 65 was chosen as the optimal value using kmergenei as suggested in Fig. 9. After assembly, contigs shorter than 200 bp were removed to generate a filtered dataset for scaffolding. Genome assembly gap filling and polishing on merged assembly was done by GapFiller v1.10^25,26^ and Pilon v1.22^27^ respectively.

**Table 4:** Summary of raw data used for *T*. *indica* hybrid assembly.

Data type	Amount of data (Mb)	Raw reads number	Total length (bp)	Average read length (bp)	Coverage depth
PacBio reads	1100	8,45,967	1,091,426,619	16,178	27.5X
Illumina reads	5300	5,323,041,232	29,900,000	100	132X

^*^Information for raw data obtained from Kumar *et al*., 2017.

**Figure 10:**
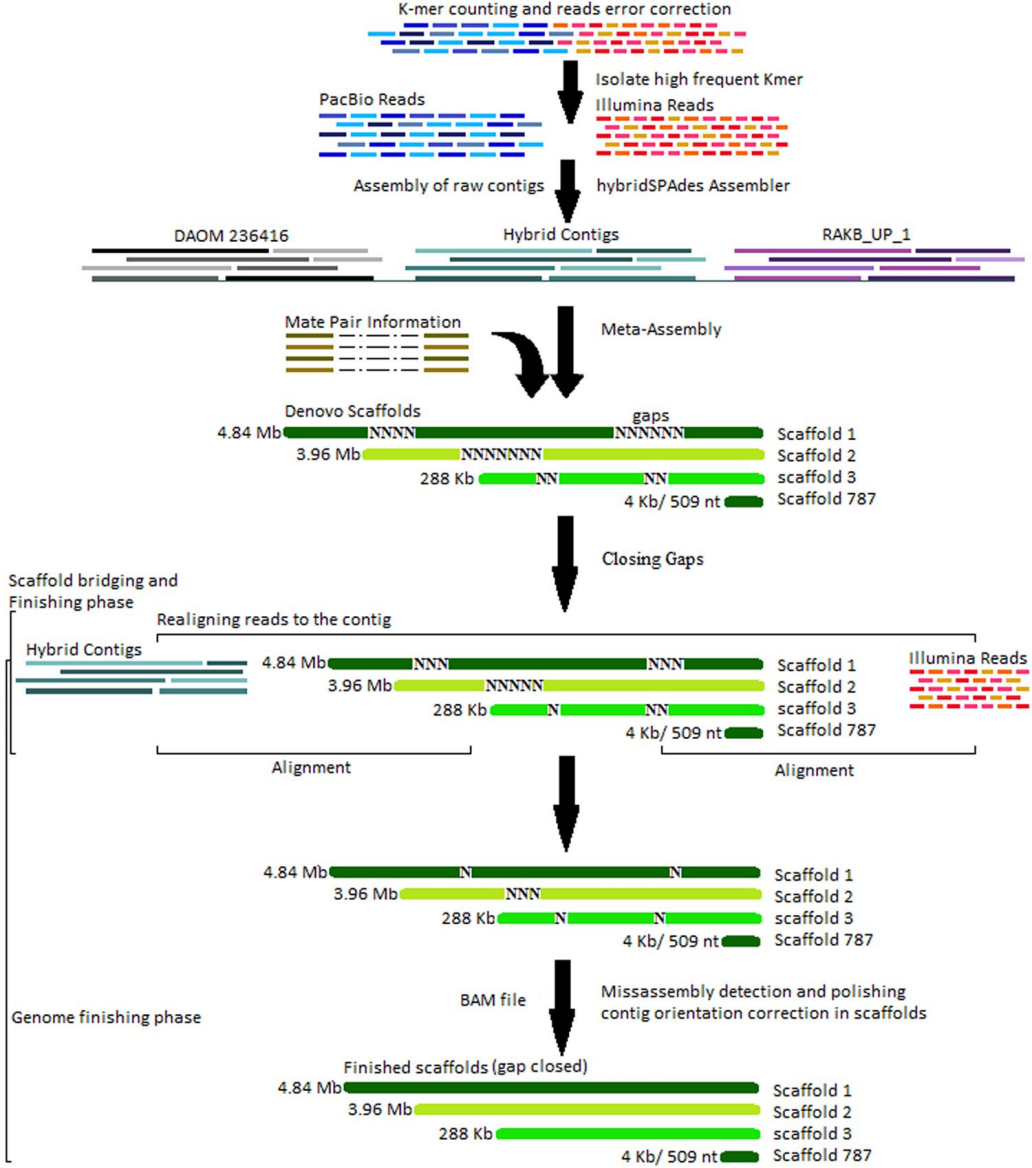
Illustration of the algorithm steps in genome reconstruction process to improve genome contiguity.

Completeness and correctness of genic sequences in *T*. *indica* assemblies are of paramount importance for direct comparison of assembled sequences^28^. However, the improved draft version of assembly was generated by using an iterative merging strategy of Metassembler v1.5^29^ by merging the inter-species draft monoteliosporic sequence-based assemblies from DAOM 236416 and RAKB_UP_1 isolates with the improved and reassembled hybrid assembly of TiK. For parameters, default values suggested in manuals were used. We included linking information from the mate pair reads to form the final set of scaffolds. Subsequently, to evaluate the accuracy of closed gaps^30^, we performed the final scaffold bridging and achieved by realigning the reads to the contigs. One more step for genome assembly gap filling and polishing with correcting the mis-assembly contig orientation on merged assembly was done by GapFiller v1.10^24,25^ and Pilon v1.22^26^ respectively (Fig. 10).

### Assembly quality assessment

High N50 value, maximum contig length and number of minimum contigs in scaffolding with respect to the high depth of coverage^31^ leads to an improved genome assembly. Towards mis-assemblies detection the number of N’s or gaps were measured, which usually result from repeats. They should be low for high quality assemblies. To achieve this, Quast v4.5^32^ was used to gather extensive assembly statistics. Completeness of set of predicted genes in genome assembly and comparison was quantitatively assessed by employing computational pipeline BUSCO v3^33^ with the latest fungal-specific orthologue catalogue. Finally, we applied the Feature-Response Curves (FRC) v1.3.0 method to evaluate the contiguity and correctness of our assembly, which is based on a prediction of assembly correctness by identifying each *de novo* assembled scaffold, where features represent the potential errors or complications during the assembly process.

### Comparative gene prediction of *T*. *indica* assemblies

The improved draft scaffolds were first repeat masked with RepeatMasker v4.0.7^34^. *De novo* gene prediction of all the genome sequences was performed with the *ab-initio* methods MAKER v2.31.9^35^, AUGUSTUS^36–38^ and SNAP^39^ which was trained with gene models of *Ustilago maydis*. To functionally annotate the predicted genes, their protein sequences were used as queries in a BLASTP search (BLAST v2.7.1) against a local installation of NCBI (http://www.ncbi.nlm.nih.gov/) non redundant protein database with an e-value cut off at 1e-6. Blast2GO v3.0 was then used to attribute GO terms and functional descriptions by performing the standard steps of InterPro search. The tRNA-encoding genes were identified by tRNAscan. In order to find pathogenicity genes, the proteins with a signal peptide were identified by SignalP v4.1 (http://www.cbs.dtu.dk/services/SignalP/) and confirmed with TargetP v1.1 (http://www.cbs.dtu.dk/services/TargetP/) were classified as for prediction of the secreted. Refined secretory proteins were filtered to have a transmembrane helix domain using TMHMM v2.0 (http://www.cbs.dtu.dk/services/TMHMM/) or a presence of GPI-anchor with PredGPI (http://gpcr.biocomp.unibo.it/predgpi/). Proteins have no transmembrane domain and one transmembrane domain within the N-terminal signal peptide was selected. Cysteine content of predicted secretory proteins was analyzed. Genes predicted to encode carbohydrate-active enzymes (CAZymes) were identified with dbCAN v4.0^40^ (http://csbl.bmb.uga.edu/dbCAN/) based on the CAZy database in the *T*. *indica* secretory proteins. To find potential virulence associated genes, a whole genome BLAST analysis was conducted against the pathogen-host interaction (PHI) gene database^41^ (http://www.phi_base.org/), a collection of genes proven to affect the outcome of pathogen-host interactions from fungi.

### Orthology and phylogenetic relationship to closely related basidiomycetes fungi

For comparing genome organization in different species it is necessary to distinguish orthologs. Ortholog cluster and gene family analysis was performed using Orthovenn^42^ on all the predicted proteomes of *Tilletia indica* TiK_1 https://www.ncbi.nlm.nih.gov/assembly/GCA_002997305.1/, *Tilletia caries* (DAOM 238032) https://www.uniprot.org/proteomes/UP000077671/, *Tilletia walkeri* (DAOM 236422) https://www.uniprot.org/proteomes/UP000078113/, *Tilletia controversa* (DAOM 236426) https://www.uniprot.org/proteomes/UP000077684/, *Tilletia horida* (QB-1) https://www.ncbi.nlm.nih.gov/assembly/GCA_001006505.1/ and *Ustilago maydis* 521 https://www.uniprot.org/proteomes/UP000000561/. An alignment was performed with threshold e-value 1 × 10^−6^ and 1.5 Inflation value. Phylogenetic profiling of *T*. *indica* with basidiomycetes fungi involved the comparison of phylogenetic data across the gene families by constructing phylogenetic trees. The comparison was performed to determine that which families have correlated or coupled evolution through which the assumptions of *Tilletia* f. spp. are functionally related can be proved. For this alignment and phylogenetic profiling were performed by using inbuilt MUSCLE algorithm of program MEGA v7.0.26^43^.

### Prediction of transposable elements and simple sequence repeats (SSRs)

Transposable elements (TEs) are mobile DNA that replicates in their host genome by new insertions and duplication at individual genomic regions and in certain instances are linked to pathogenicity. TE insertions often result in increased number of coded genes and thereby play significant role in enhancing fungal pathogenesis^44^. It may have a prominent role in the regulation of gene expression^45^. *T*. *indica* genome were subjected to RepeatMasker for *de novo* repeat prediction and it was run with the –s (sensitive) setting. Reference based repeats finding was performed by comparing to reference repeats library of RepBase database (http://www.girinst.org/repbase/) and were annotated with TranposonPSI (http://transposon.sourceforge.net). Subsequently, the whole genome sequence of *T*. *indica* was subjected to find the distribution and frequency of various SSRs types using Microsatellite Identification tool (MISA) (http://pgrc.ipkgatersleben.de/misa/). Minimum length for SSR motifs per unit size was set to 10 for mono, 6 for di and 5 for a tri, tetra, penta, hexa motifs. We calculated the total lengths of all mono-, di-, tri-, tetra-, penta-, and hexa-nucleotide repeats in terms of base pairs of SSR per megabase pair (Mb) of DNA. ORF finder (NCBI) was employed to find total number of Open reading frames (ORFs) present among the analyzed *T*. *indica* isolates.

### Identification of SNPs/InDels for variant analysis

To assess the variation present among analyzed field isolates SNPs and indels were identified in the genome data. Sequencing reads from isolates were first mapped to the genome of reference assembly, using Bowtie and then the sorted alignment BAM files were inputted to GATK’s UnifiedGenotyper^46^ (https://software.broadinstitute.org/gatk/) and Varscan^47^ (http://varscan.sourceforge.net/) for SNP identification. To identify indels, a pipeline including SAMtools and BCFtools (http://samtools.sourceforge.net/mpileup.shtml) was used to process alignment files. All identified indels were supported by at least 8 reads. Then annotation of SNPs/indels was performed by SnpEff based available gene sets.

### Computational resources

We run all assembly and merging using Linux platform Ubuntu-16.04-xenial version on a dell Precision Model T7500 workstation having 2.8 GHz Intel Xeon Octa-Core processors and 48 GB of RAM. Majority of the running time is spent on assembly process and about 1/4 on graph construction and analysis. However, Reconciliator uses about 21 h and more than 40GB of RAM to merge the TiK isolate, RAKB_UP_1 and DAOM 236416 assemblies.

## Supplementary information

Supplementary Data 3

Supplementary Data 1-2 and 4-5

## Data Availability

The present improved whole-genome shotgun project has been deposited at DDBJ/ENA/GenBank, under the Aaccession Number PKQB00000000. The version described in this report is version PKQB01000000.

## References

[1] Mitra M (1931). A new bunt on wheat in India. Annals of Applied Biology.

[2] Kumar A, Singh US, Kumar J, Garg GK (2008). Application of molecular and immuno-diagnostic tools for detection, surveillance and quarantine regulation of Karnal bunt (*Tilletia indica*) of wheat. Food and Agricultural Immunology.

[3] Gupta AK, Seneviratne JM, Bala R, Jaiswal JP, Kumar A (2015). Alteration of genetic make-up in Karnal Bunt pathogen *(Tilletia indica*) of wheat in presence of host determinants. Plant Pathology Journal.

[4] Kumar A, Singh US, Singh A, Malik VS, Garg GK (2000). Molecular signalling in pathogenicity and host recognition in smut fungi taking Karnal bunt as a model system. Indian Journal of Experimental Biology.

[5] Dhaliwal HS (1989). Multiplication of secondary sporidia of *Tilletia indica* on soil and wheat leaves and spikes and occurrence of Karnal bunt. Can. J. Bot..

[6] Nagarajan S (1997). Karnal bunt (*Tilletia indica*) of wheat—A review. Rev. Plant Pathol..

[7] Singh RA, Krishna A (1982). Susceptible stage for inoculation and effect of Karnal bunt on viability of wheat seed. Indian Phytopathol..

[8] Goates BJ (1988). Histology of infection of wheat by *Tilletia indica*, the Karnal bunt pathogen. Phytopathology.

[9] Rush CM (2005). Status of Karnal Bunt of Wheat in the United States 1996 to 2004. Plant Disease.

[10] Kumar A (2017). Draft genome sequence of Karnal bunt pathogen (*Tilletia indica*) of wheat provides insights into the pathogenic mechanisms of quarantined fungus. PLoS ONE.

[11] Nguyen, H. D., Samba, S. P., Cullis, J., Levesque, C.A. & Hambleton, S. Draft genome sequence of *Tilletia indica* and *Tilletia walkeri*. Submitted (APR-2016) to the EMBL/GenBank/DDBJ databases (accession no. GCA_001645015.1) (2016).

[12] Aggarwal, R. *et al*. Data from GenBank (accession no. GCA_002220835.1) (2017).

[13] Thomma BPHJ (2016). Mind the gap; seven reasons to close fragmented genome assemblies. Fungal Genetics and Biology.

[14] Fraser CM, Eisen JA, Nelson KE, Paulsen IT, Salzberg SL (2002). The Value of Complete Microbial Genome Sequencing (You Get What You Pay For). Journal of Bacteriology.

[15] Kumar A (2018). Improved draft genome sequence of a monoteliosporic culture of the Karnal bunt (*Tilletia indica*) pathogen of wheat. Genome Announcement.

[16] Kumar A, Singh US, Singh A, Malik VS, Garg GK (2000). Molecular signaling in pathogenicity and host recognition in smut fungi taking Karnal bunt as a model system. Indian Journal of Experimental Biology.

[17] Hittalmani S, Mahesh HB, Mahadevaiah C, Prasannakumar MK (2016). *De novo* genome assembly and annotation of rice sheath rot fungus *Sarocladium oryzae* reveals genes involved in Helvolic acid and Cerulenin biosynthesis pathways. BMC Genomics.

[18] Gladieux P (2014). Fungal evolutionary genomics provides insight into the mechanisms of adaptive divergence in eukaryotes. Molecular Ecology.

[19] Nowrousia M (2010). Next-Generation Sequencing Techniques for Eukaryotic Microorganisms: Sequencing-Based Solutions to Biological Problems. Eukaryotic Cell.

[20] Kumar A, Singh A, Garg GK (1998). Development of Seed Immunoblot Binding Assay for Detection of Karnal bunt (*Tilletia indica*) of Wheat. Journal of Plant Biochemistry and Biotechnology.

[21] Sharma P (2016). Draft genome sequence of two monosporidial lines of the Karnal bunt fungus *Tilletia indica* Mitra (PSWKBGH-1 and PSWKBGH-2). Genome Announcement.

[22] Antipov D, Korobeynikov A, McLean JS, Pevzner P (2016). A. hybridSPAdes: an algorithm for hybrid assembly of short and long reads. Bioinformatics.

[23] Desai A (2013). Identification of optimum sequencing depth especially for *de novo* genome assembly of small genomes using next generation sequencing data. PLoS ONE.

[24] Chikhi R, Medvedev P (2014). Informed and automated k-mer size selection for genome assembly. Bioinformatics.

[25] Nadalin F, Vezzi F, Policriti A (2012). GapFiller: a *de novo* assembly approach to fill the gap within paired reads. BMC Bioinformatics.

[26] Boetzer M, Pirovano W (2012). Toward almost closed genomes with GapFiller. Genome Biology.

[27] Walker BJ (2014). Pilon: An Integrated Tool for Comprehensive Microbial Variant Detection and Genome Assembly Improvement. PLoS ONE.

[28] Kremer FS, McBride AJA, Pinto LDS (2017). Approaches for in silico finishing of microbial genome sequences. Genetics and Molecular Biology.

[29] Wence AH, Schatz MC (2015). Metassembler: merging and optimizing *de novo* genome assemblie. Genome Biology.

[30] Utturkar SM, Klingeman DM, Hurt RA, Brown SD (2017). A Case Study into Microbial Genome Assembly Gap Sequences and Finishing Strategies. Frontiers in Microbiology.

[31] Sims D, Sudbery I, Ilott NE, Heger A, Ponting CP (2014). Sequencing depth and coverage: key considerations in genomic analyses. Nature Reviews.

[32] Gurevich A, Saveliev V, Vyahhi N, Tesler G (2013). QUAST: quality assessment tool for genome assemblies. Bioinformatics.

[33] Simão FA, Waterhouse RM, Ioannidis P, Kriventseva EV, Zdobnov EM (2015). BUSCO: assessing genome assembly and annotation completeness with single-copy orthologs. Bioinformatics.

[34] Tarailo-Graovac M, Chen N (2009). Using Repeat Masker to Identify Repetitive Elements in Genomic Sequences. Current protocol in Bioinformatics.

[35] Cantarel BL (2008). MAKER: An easy-to-use annotation pipeline designed for emerging model organism genomes. Genome Research.

[36] Stanke M, Schoffmann O, Morgenstern B, Waack S (2006). Gene prediction in eukaryotes with a generalized hidden markov model that uses hints from external sources. BMC Bioinformatics.

[37] Stanke M, Morgenstern B (2005). AUGUSTUS: a web server for gene prediction in eukaryotes that allows user defined constraints. Nucleic Acid Research.

[38] Stanke M, Steinkamp R, Waack S, Morgenster B (2004). AUGUSTUS: a web server for gene finding in eukaryotes. Nucleic Acid Research.

[39] Korf I (2004). Gene finding in novel genomes. BMC Bioinformatics.

[40] Yanbin Y (2012). dbCAN: a web resource for automated carbohydrate-active enzyme annotation. Nucleic Acids Res..

[41] Urban M (2015). The Pathogen-Host Interactions database: additons and future developments. Nucleic Acids Research.

[42] Wang Y, Coleman-Derr D, Chen G, Gu YQ (2015). OrthoVenn: a web server for genome wide comparison and annotation of orthologous clusters across multiple species. Nucleic Acids Research.

[43] Kumar, S., Stecher, G. & Tamura, K. MEGA7: Molecular Evolutionary Genetics Analysis version 7.0 for bigger datasets. *Molecular Biology and Evolution* (2016). 10.1093/molbev/msw054 PMC8210823 27004904

[44] Baidouri ME (2015). A new approach for annotation of transposable elements using small RNA mapping. Nucleic Acids Research.

[45] Vitte C, Fustier MA, Alix K, Tenaillon MI (2014). The bright side of transposons in crop evolution. Briefings in Functional Genomics.

[46] McKenna A (2010). The Genome Analysis Toolkit: a MapReduce framework for analyzing next-generation DNA sequencing data. Genome Research.

[47] Koboldt D (2012). VarScan 2: Somatic mutation and copy number alteration discovery in cancer by exome sequencing. Genome Research.

