# Supplementary material for: Comparative genomic analysis of monosporidial and monoteliosporic cultures for unraveling the complexity of molecular pathogenesis of Tilletia indica pathogen of wheat
Source: Sci Rep. 2019 Jun 3;9:8185. doi: 10.1038/s41598-019-44464-0 (PMC6547692; doi:10.1038/s41598-019-44464-0)
Supplement: Supplementary file 2 — Supplementary Data 1-2 and 4-5 [file 41598_2019_44464_MOESM2_ESM.pdf]

Comparative genomic analysis of monosporidial and monoteliosporic cultures for unraveling the complexity of molecular pathogenesis of *Tilletia indica* pathogen of wheat

Pallavi Mishra[a,b,c#], Ranjeet Maurya[a#], Vijai K. Gupta[d], Pramod W. Ramteke[e*], Soma S. Marla[b*], Anil Kumar[a,f*]

AUTHOR AFFILIATIONS

[a]Department of Molecular Biology and Genetic Engineering, College of Basic Sciences and Humanities, G. B. Pant University of Agriculture and Technology, Pantnagar, India.

[b]Division of Genomic Resources, ICAR-National Bureau of Plant Genetic Resources, New Delhi, India.

[c]Department of Computational Biology and Bioinformatics, Sam Higginbottom University of Agriculture, Technology and Sciences, Allahabad, India.

[d]Department of Chemistry and Biotechnology, ERA Chair of Green Chemistry, Tallinn University of Technology, Tallinn, Estonia.

[e]Department of Biological Sciences, Sam Higginbottom University of Agriculture, Technology and Sciences, Allahabad, India.

[f] Rani Lakshmi Bai Central Agricultural University, Jhansi, India.

* To whom correspondence should be addressed: **anilkumar.mbge@gmail.com, pwramteke@gmail.com, soma.marla@icar.gov.in**

Supplementary Data 1

Supplementary Data 1: The frequency of the most common motif appears predominatly is displayed for each developmental case.

Predominant repeats	monoteliosporic TiK_1	Monosporidial PSWKBGH_1	Monosporidial PSWKBGH_2	Dikaryon PSWKBGD_1_3
**AG/CT**	1131	1217	1163	1388
**A/T**	668	1049	963	1130
**C/G**	641	1113	1192	1763
**AGC/CTG**	516	600	537	620
**AGG/CCT**	494	562	536	640
**AC/GT**	412	416	472	487
**ACC/GGT**	337	347	376	378
**AAC/GTT**	263	272	270	321
**AAG/CTT**	223	253	252	298
**CCG/CGG**	203	259	197	245
**ATC/ATG**	198	199	200	261
**ACG/CGT**	190	277	227	268
**CG/CG**	52	64	46	58
**ACTC/AGTG**	50	55	59	68
**AAGG/CCTT**	27	32	30	41
**ACT/AGT**	22	19	33	31
**AT/AT**	18	18	12	20
**AGGG/CCCT**	18	35	27	18
**AACCCT/AGGGTT**	12	39	73	15
**AAAG/CTTT**	8	10	44	22
**ACAG/CTGT**	8	50	14	13

Supplementary Data 2

Supplementary Data 2: Protein family annotation shows the distribution of most abundant protein class assigned to *T. indica* genes.

Category name (Accession)	Genes	Percent of gene hit against total genes	Percent of gene hit against total Protein Class hits
extracellular matrix protein (PC00102)	4	0.10%	0.20%
cytoskeletal protein (PC00085)	58	1.90%	2.80%
transporter (PC00227)	148	4.80%	7.20%
transmembrane receptor regulatory/adaptor protein (PC00226)	9	0.30%	0.40%
transferase (PC00220)	281	9.10%	13.70%
oxidoreductase (PC00176)	197	6.40%	9.60%
lyase (PC00144)	60	1.90%	2.90%
cell adhesion molecule (PC00069)	1	0.00%	0.00%
ligase (PC00142)	98	3.20%	4.80%
nucleic acid binding (PC00171)	408	13.20%	19.90%
signaling molecule (PC00207)	14	0.50%	0.70%
enzyme modulator (PC00095)	125	4.00%	6.10%
calcium-binding protein (PC00060)	27	0.90%	1.30%
defense/immunity protein (PC00090)	1	0.00%	0.00%
hydrolase (PC00121)	332	10.70%	16.20%
transfer/carrier protein (PC00219)	45	1.50%	2.20%
membrane traffic protein (PC00150)	69	2.20%	3.40%
transcription factor (PC00218)	81	2.60%	3.90%
chaperone (PC00072)	25	0.80%	1.20%
cell junction protein (PC00070)	2	0.10%	0.10%
structural protein (PC00211)	2	0.10%	0.10%
isomerase (PC00135)	40	1.30%	1.90%
receptor (PC00197)	24	0.80%	1.20%

Supplementary Data 4

Supplementary Data 4: Virulence associated genes (VAGs) belonging to T. indica genome sequence producing high-scoring segments pairs.

**S.No.**	**Gene phenotype**	**Number of VAGs**
1	loss_of_pathogenicity	33
2	unaffected_pathogenicity	50
3	reduced_virulence	90
4	lethal	22
5	increased_virulence (hypervirulence)	4
6	resistance_to_chemical	1
7	multiple outcome	20
	**Total VAGs**	**220**

Supplementary data 5

Supplementary data 5: The PHI-base accessions assigned to *T. indica* genes hits to other pathogenic fungal organism.

**Organisms**	**No. of hits of *T. Indica***
Fusarium_graminearum	58
Magnaporthe_oryzae	34
Cryptococcus_neoformans	24
Ustilago_maydis	20
Aspergillus_fumigatus	19
Candida_albicans	12
Parastagonospora_nodorum	8
Colletotrichum_gloeosporioides	6
Cryptococcus_gattii	4
Colletotrichum_lindemuthianum	3
Edwardsiella_ictaluri	3
Leptosphaeria_maculans	3
Saccharomyces_cerevisiae	3
